# Will Cardiovascular Disease Prevention Widen Health Inequalities?

**DOI:** 10.1371/journal.pmed.1000320

**Published:** 2010-08-24

**Authors:** Simon Capewell, Hilary Graham

**Affiliations:** 1 Department of Public Health, University of Liverpool, Liverpool, United Kingdom; 2 Department of Health Sciences, University of York, Heslington, York, United Kingdom

## Abstract

Simon Capewell and Hilary Graham review different population strategies for preventing cardiovascular disease and conclude that screening and treating high-risk individuals may be ineffective and widen social inequalities.

**Summary Points**

- The primary prevention of cardiovascular disease (CVD) is dependent on the effective reduction of the major risk factors for CVD, particularly tobacco control and a healthier diet.
- The high-risk approach to prevent CVD typically involves population screening. Those exceeding a risk threshold are then given lifestyle advice and/or tablets to reduce blood cholesterol and blood pressure.
- Evidence suggests this high-risk approach typically widens socioeconomic inequalities. Such inequalities have been reported in screening, healthy diet advice, smoking cessation, statin and anti-hypertensive prescribing, and adherence.
- The alternative approach is population-wide CVD prevention. For example, legislating for smoke-free public spaces, banning dietary transfats, or halving daily dietary salt intake. Such strategies are generally effective and cost-saving; there is also increasing evidence that they can reduce health inequalities.
- We conclude that screening and treating high-risk individuals represents a relatively ineffective CVD prevention approach that typically widens social inequalities.

## Introduction

Several high-income countries, including the United Kingdom, are tackling “health inequalities” [1]. In 2009, the various UK governments announced large-scale programmes to screen and treat cardiovascular risk [2]. The respective health ministers stated that the programmes would reduce health inequalities, although opposition parties generally predicted the opposite [3]. The potential effects of any screening policy on health inequalities clearly need to be urgently considered, not least in order to inform current policy development in the UK [4],[5] and internationally [6].

The primary prevention of cardiovascular disease (CVD) is dependent on the effective reduction of the major risk factors, particularly by reducing tobacco use and adopting a healthier diet [2]. However, the substantial excess burden of morbidity and mortality due to CVD in disadvantaged groups raises major challenges. Social gradients in the major cardiovascular risk factors can explain approximately three-quarters of this excess burden; smoking alone can explain more than half [7],[8].

Assessing the potential effect of risk factor reductions on socioeconomic inequalities in health is crucial. McLaren et al. usefully distinguish between “agentic” prevention strategies (which rely solely on individuals making and sustaining behaviour change) and “structural” strategies (which work through changes in the wider social environment [9]. There is increasing evidence to suggest that addressing CVD risk factors using “structural” whole-population approaches generally reduces social inequalities. There is also worrying preliminary evidence that screening and treating high-risk individuals (“agentic” strategies) might increase the inequalities gap. In this Policy Forum article, we review this evidence, and consider different potential approaches for reducing inequalities.

## The Whole-Population Approach for Preventing CVD

Some two decades ago, Geoffrey Rose suggested that a small reduction in risk in a large number of people may prevent many more cases than treating a small number at higher risk [10]. He therefore cautioned against simply pursuing individual-level interventions targeted at changing risk profiles in this latter group. Rose instead advocated a dual strategy, also using a whole-population approach to change everyone's exposure. That approach would support policies that work directly on what Rose called “the underlying causes of disease”; for example, via statutory regulation and environmental controls, rather than indirectly by changing risk factors on a person-by-person basis. Whole-population interventions can indeed reduce risk factors across entire countries. National legislation and fiscal policies can be both effective and cost-saving, whether banning industrial transfats (Denmark), halving dietary salt in processed foods (Finland), or promoting smoke-free public spaces (Scotland, Ireland, Italy, and elsewhere) [11]–[14].

Growing international evidence now supports the Rose hypothesis [15]–[17]. Small reductions in population cholesterol concentrations, blood pressure, or smoking then translate into substantial reductions in cardiovascular events and deaths [17]–[19]. This evidence suggests that comprehensive policies can be more effective in reducing risk factors and improving health than a high-risk individual approach. Furthermore, identifying individuals with a threshold of a 20% 10-year CVD event risk would then necessitate multiple preventive treatments for one-quarter of the population. In the UK, this might decrease UK cardiovascular mortality by approximately 17% (assuming normal adherence). Conversely, country-wide policies to reduce cholesterol and smoking population levels by just 5% would decrease UK mortality substantially more, by about 26% [15]. Capewell et al. reported similar findings for the US population [18].

## The Whole-Population Approach for Reducing Social Inequalities in CVD

There is increasing evidence to support health equity strategies that take a whole-population approach to CVD risk factors. This includes simply considering arithmetical principles. *Disadvantaged groups experience a greater CVD burden. They are thus likely to gain extra benefit if a risk factor is uniformly reduced across the entire population, with a consequent reduction in absolute (but not necessarily relative) inequalities.* This simple arithmetic was spelt out by Diederichsen and colleagues [20].

More recent support came from Kivimaki et al., who quantified the 15-year benefits of decreasing risk factors uniformly across a male population (reductions of 10 mmHg in blood pressure, 2 mmol/l in total cholesterol, and 1 mmol/l in glucose) [21]. Although relative inequalities would remain, such interventions might reduce the absolute mortality gap between rich and poor by approximately 70% [21].

Smoking rates and exposure to environmental tobacco smoke are higher in poorer groups in Scotland, which is consistent with other high-income countries [22]. However, following the Scottish smoke-free legislation in 2006, there was a substantial fall in hospital admissions for heart attack and “acute coronary syndrome” (involving a 14% reduction in smokers and a 21% fall in never smokers). This drop was uniform across social groups [13].

Strong regulatory policies, particularly those including increases in cigarette price, are also associated with declines in tobacco use of a similar magnitude across socioeconomic groups [23]. This suggests that, in the many countries where smoking rates are higher in poorer groups, the absolute benefit will be greater than in affluent groups. Indeed, men and women in lower socioeconomic groups appear more responsive to uniform increases in cigarette price than affluent groups [24],[25]. However, attention needs to be paid to how inequalities within disadvantaged groups can influence responses to population-wide interventions and their overall impacts [26].

Social differences are observed in diet, as in smoking. Thus, low-income families consume more saturated fat and fewer fruits and vegetables than more affluent families [27]. Strong supporting evidence for the effectiveness of a population-wide diet intervention comes from the United States. Folic acid fortification of cereals was introduced in 1996. Absolute social differences in blood folate levels were subsequently reduced by 67% [28]. Furthermore, comparable reductions in inequalities in dental caries followed water fluoridation [29]. The implications are clear. Eradication of dietary transfats, or halving the salt content of bread, would disproportionately benefit deprived groups.

Of course, the population approach is unlikely to totally abolish inequalities since many of the drivers of disadvantage lie even further upstream. For instance, structural interventions in the Ontario Smoke Free Strategy included smoking bans in enclosed public places and enclosed work places, laws on tobacco sales to minors, and restrictions on the display of tobacco products in retail outlets. Overall smoking rates in the province fell. However, 40% of aboriginal women and men are still smoking, as are 34% of adults with less than a secondary school education compared to 11% who had a bachelor's degree or higher [30].

The population approach has a strong ethical base. It is in step with the “stewardship” model of public health that places obligations on governments to enable conditions in which everyone can lead a healthy life [31]. Classic examples include legislating for clean drinking water, seatbelts, and food hygiene. Such principles have long underpinned broader policies to protect well-being, by regulating market economies and providing for basic needs [32]. There is also some support from the political right under the banner of “libertarian paternalism” or “nudge” (routinely presenting options to increase the likelihood that people will choose what they would on reflection most prefer) [33].

However, population-based structural approaches to reduce inequalities might be difficult to achieve. Such approaches ideally require concerted cross-sectoral efforts such as universal access to healthy food, reductions in work place stress, and access to safe environments for physical activity for all [32].

## The High-Risk Approach for Preventing CVD

In the UK, the high-risk approach for preventing CVD is typified by the health checks programme Putting Prevention First, implemented in England [2]. All adults aged 40–74 years will be invited to be screened for CVD risk. Individuals found to exceed a 20% risk of a cardiovascular event in the next 10 years will be treated with a combination of lifestyle advice plus tablets to reduce blood cholesterol and blood pressure, as appropriate [2].

This is a controversial area. Manuel et al. recently “revisited” Rose [34]. Their influential article advocated the high-risk approach [34]. However, their methodology and conclusions were subsequently criticised by Whincup and others [35]. The methodological limitations identified by these critics meant that firstly, the Manuel analysis systematically over-estimated the likely benefit of individual strategies (by including patients with established CVD, inflating the numbers in the “high-risk” group, assuming that effectiveness in routine clinical practice equalled efficacy in RCTs, and ignoring under-treatment and poor long-term adherence). Secondly, they systematically under-estimated the contribution of population strategies (by conservatively assuming a 2% reduction in population cholesterol when falls of 10%–18% have been observed elsewhere, and by using an unvalidated model and also failing to mention that population approaches to prevention also reduce the pool of high-risk people requiring drug treatment) [35].

Likewise, Zulman et al. recently preferred a high-intensity treatment intervention in the US adult population [36]. However, their mortality estimates were 3-fold higher than previous publications [36]. This over-estimate probably reflected successive optimistic assumptions about effectiveness and long-term adherence [36],[37].

Furthermore, critics of the high-risk cardiovascular risk screening approach suggest that this strategy might have low effectiveness, leave substantial residual risk, and achieve a small population impact at high cost; as well as result in the medicalisation of previously healthy individuals. Furthermore, it does not address the root causes of the problem [38]–[40]. Equally seriously, this high-risk approach will almost certainly widen inequalities.

## The High-Risk Approach May Worsen Social Inequalities in CVD

There is increasing evidence that inequalities in risk factors can widen when effects are mediated through individual-level changes in knowledge, motivation, and behaviour (for example, national health promotion campaigns and behavioural change programmes) [41],[42]. Furthermore, because such interventions do not work directly on population exposure to risk factors, they do not address inequalities in risk-factor profiles in subsequent cohorts.

“Agentic” interventions, which require mobilisation of an individual's resources, whether material or psychological, generally favour those with more resources, thus tending to increase social inequalities [9],[41],[42]. This parallels what Tudor Hart memorably described as the “Inverse Care Law”**—**the availability of good medical care tends to vary inversely with the need for it in the population served [43]. Thus, the people in the poorest health gain the lowest net health benefit from the interventions [43]. Disadvantage can occur at every stage in the process, from the person's beliefs about health and disease, and actual health behaviour, to presentation, screening, risk assessment, negotiation, participation, programme persistence, and treatment adherence. Tugwell et al. usefully described this cumulative inequality as the “staircase effect” [44].

Inequalities have also been reported in the screening and detection of cancer as well as CVD. For instance, women who choose to attend the National Health Service (NHS) Breast Screening Programme come more from affluent areas [45].

In the US, Frohlich's analysis likewise suggested that even when individual-based interventions are widely applied (such as screening or health information campaigns), they may increase disparities [46]. Furthermore, examples of the inverse care law in CVD primary prevention prescribing have also been reported. Substantial socioeconomic gradients exist in statin use, both in the UK and in the Danish health care system, which aims, like the NHS, to ensure equity in medical care [47]–[49].

Likewise, inequalities in anti-hypertensive therapy have been reported. A recent study suggested that social and ethnic disparities in the detection and management of hypertension have persisted in the UK despite major investment in quality improvement initiatives, including pay for performance [50]. Long-term adherence (compliance) with primary prevention medications barely reaches 50%, and is often worse in more deprived groups [51]–[53]. Furthermore, inequalities in adherence have been specifically reported for both statins and anti-hypertensive medications [54],[55].

For smoking cessation, greater use and higher quit rates of cessation services by more advantaged individuals are a real concern [56]. Affluent smokers tend to receive more help, and are more likely to quit [57],[58]. Increasing quit rates in more affluent smokers were also recently reported in Inter99, the Danish trail of primary prevention in general practice [59]. Similar inequalities have also been reported in workplace smoking interventions [57].

With respect to dietary advice, US policies traditionally favour individual approaches over public health strategies. There, Kanjilal and colleagues recently reported bigger declines in CVD risk factors in more affluent groups [60]. Supporting evidence comes from a recent systematic review of nutritional interventions in individuals and groups [61]. In schools, fruit and vegetable consumption typically increased more in affluent families; interventions were correspondingly less effective in disadvantaged areas. Likewise, in a US primary care setting, interventions to reduce fat intake were less successful in blacks than in (more affluent) whites [61]. In Germany, the Cardiovascular Prevention Study compared three strategies involving advice from professionals and media. After 7 years, hypercholesterolaemia improved only in upper social groups, thereby increasing the gap between the health of rich and poor [62].

In England, a high-risk approach to CVD prevention that specifically prioritises disadvantaged groups and localities is being actively promoted. The National Institute for Health and Clinical Excellence recently published public health guidelines advising specific approaches for identifying and supporting people most at risk of dying prematurely [63]. Elsewhere, more innovative strategies are being developed for poor communities—for example, use of non-physician health care workers, financial incentives, and availability of low-cost generic “polypills” [64],[65]. Evidence to confirm the effectiveness and cost-effectiveness of such targeted strategies in reducing health inequalities is currently being gathered [66]. Results are eagerly awaited.

## Combining the Population-Based and High-Risk Approaches?

Might a coordinated approach that integrates population-based and high-risk approaches be more effective? The Norsjo Community Intervention Program in Sweden is an example of a model that combines population health and health sector interventions. The program created a local health promotion collaboration between healthcare providers, grocery stores, schools, and municipal authorities. Primary care physicians contacted patients for systematic risk factor screening and counselling aimed at CVD risk reduction. Community interventions included changes in food labelling to make it easier to adhere to dietary recommendations. The predicted CVD mortality risk was reduced by 36% in the intervention area compared to 1% in a control community. Socioeconomically less privileged groups benefited more from the program [67].

## Specifically Targeting High-Risk Populations?

Socioeconomically disadvantaged populations are susceptible to under-diagnosis of hypertension, diabetes, and hypercholesterolemia and also to suboptimal care for interventions to reduce risk. Risk factor modification through tailored interventions in high-risk groups might therefore produce considerable benefits; however, evaluation is urgently required.

## Conclusions

Given the ubiquity of social and health inequalities, we should not be surprised if interventions to reduce CVD have differential effects, with advantaged groups deriving greater benefit than poorer groups. We have suggested that the potential for such unequal effects is greater for high-risk approaches, where change is contingent on action by individual patients and healthcare providers, compared with whole population approaches, where change is societal and instituted collectively by agencies with statutory responsibility for public health.

Operating mainly outside the health service, the population approach offers governments the opportunity to act directly on population exposure to risk factors. It thus addresses the major drivers of health and health inequalities [68]. Meanwhile, evidence that healthcare interventions can generate and compound risk-factor inequalities is steadily accumulating [42]. We therefore look forward to future analyses from Tugwell and other colleagues in the Cochrane Health Equity Field [44]. However, that is no excuse for delay.

In conclusion, there is evidence that CVD prevention strategies for screening and treating high-risk individuals may represent a relatively ineffective approach that typically widens social inequalities. In contrast, policy interventions to limit risk-factor exposure across populations appear cheaper and more effective; they could also contribute to levelling health across socioeconomic groups. The two approaches are complementary, and Rose's advocacy of a dual strategy may prove prophetic [10]. However, all future strategies aimed at improving population health will merit rigorous evaluation of their potential impact on inequities.
